# An Uncomfortable Neighborhood: Presence Evolution of Two Competing Carnivores in North‐Eastern Italy

**DOI:** 10.1002/ece3.72368

**Published:** 2025-10-24

**Authors:** Lorenzo Frangini, Lorenzo Bernicchi, Marco Franchetto, Ursula Sterrer, Hanna Steigleder, Matteo Canazza, Marcello Franchini, Virginia Barca, Giovanna Miani, Andrea Madinelli, Stefano Filacorda

**Affiliations:** ^1^ Department of Agricultural, Food, Environmental and Animal Sciences University of Udine Udine Italy; ^2^ Faculty of Science and Technology Free University of Bozen‐Bolzano Bolzano Italy; ^3^ Foundation KORA – Carnivore Ecology and Wildlife Management Talgut‐Zentrum Ittigen, BE CH Switzerland; ^4^ Institute for Alpine Environment Eurac Research Bolzano/Bozen Italy; ^5^ Department of Environmental Science and Policy University of Milan Milan Italy

**Keywords:** *Canis aureus*, *Canis lupus*, distribution map, sympatry

## Abstract

Within ecological communities, larger predators typically limit mesocarnivore populations. On a continental scale, this may be the case for the gray wolf ( *Canis lupus* ) over the golden jackal ( *Canis aureus* ) in Europe. North‐eastern Italy represents one of the first areas in Western Europe to experience golden jackal colonization, followed by wolf recolonization. Since few studies have investigated the spatial relationship between these two wild canids, this work aimed to analyze their distribution dynamics and investigate whether the wolf and environmental factors may have influenced golden jackal distribution. We used systematic and opportunistic data collected over 11 years (2013–2023) to assess the presence of both species on a 10 × 10 km square grid system. A multinomial logistic mixed model (MLMM) was applied to test whether the study period and landscape metrics (terrain ruggedness and habitat fragmentation indices) influenced both species' presence at a broad scale (i.e., square grid units). Generalized linear mixed models (GLMMs) were used to test whether the wolf and landscape metrics influenced jackal presence at a finer scale (i.e., jackal howling calling stations). Both species showed an increase in their former distribution, with a small growth in sympatric areas. Models revealed that jackals preferred less rugged and more fragmented areas typical of the lowland, whereas wolves preferred rugged terrain with extensive forested patches in the Alpine range. Furthermore, our results showed that, at a local scale, golden jackal presence was negatively related to wolf presence. This study provides further insights into the coexistence of these two competing wild canids, suggesting a potential top‐down effect of the wolf on golden jackal colonization dynamics. However, the wolf influence may vary in intensity depending on environmental context, with a weaker effect in areas of higher human pressure, such as the lowlands.

## Graphical abstract

This study examined the spatial dynamics between gray wolves and golden jackals in north‐eastern Italy from 2013 to 2023, as both species expanded their ranges. Wolves favored rugged, forested Alpine areas, while jackals preferred fragmented, lowland habitats. At a local scale, jackal presence was negatively associated with wolf presence, suggesting a potential top‐down effect by wolves, though this influence may be weaker in human‐dominated landscapes.

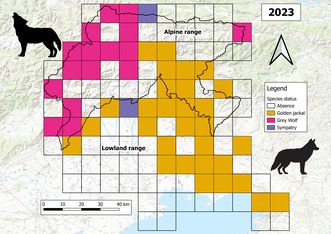

## 1 Introduction

The coexistence of species living in sympatry is of great interest in ecology. Exploring relationships among organisms helps scientists to explain the complexity of ecosystems, providing useful information to understand their functioning and, in turn, for conservation and management purposes (Ritchie and Johnson 2009; Schwenk et al. 2009). Indeed, direct and indirect animal interactions determine species distributions and abundances by shaping community structures (Schoener 1983; Rosenzweig 1995; Ritchie and Johnson 2009). Within the vertebrate community, large carnivores, defined as all carnivores weighing more than 15 kg (Ripple et al. 2014), typically act as top predators in the ecosystems, thus strongly interacting with a wide range of species (Duffy 2003). Consequently, their decline or disappearance might lead to significant—and likely detrimental—effects on ecological communities and ecosystems, as depicted by the mesopredator release hypothesis, which states that the loss of large predators may release populations of smaller predators (Crooks and Soule 1999; Ritchie and Johnson 2009). This process might have dramatic ecological effects, via predation and competition, affecting a wide range of wildlife species (Ripple et al. 2013).

The co‐occurrence of carnivore species has been widely investigated (e.g., Karanth et al. 2017; Klauder et al. 2021), and there is evidence of the negative correlation between apex predators and mesocarnivores (Berger et al. 2008; Pasanen‐Mortensen et al. 2013; Zhao et al. 2020). Indeed, apex predators are typically dominant over smaller carnivores, influencing their space and/or time use to avoid competitive/lethal encounters (Di Bitetti et al. 2010; Zhao et al. 2020). However, mesocarnivores might benefit from proximity to large carnivores due to their scavenging behavior (Allen et al. 2015; Ferretti et al. 2021) or by using them as a shield against competition or risk from other mesocarnivores (Newsome and Ripple 2015; Allen et al. 2017). However, studying species interactions is challenging, especially when carnivores are involved. These difficulties are mainly linked to their crepuscular/nocturnal activity, large home ranges, and low population densities (Balme et al. 2009). Technological advances have allowed researchers to partially fill these gaps by collecting different types of data, i.e., telemetry (Schroeder et al. 2015; Davies et al. 2021), camera‐traps (Caravaggi et al. 2017; Franchini et al. 2023), and even occurrence data from open‐access online repositories (i.e., GBIF; Jones et al. 2016). Once properly gained, such data can be used to develop fine‐scale analyses at the individual (Kittle et al. 2016; Lewis et al. 2017) or population level (Zhao et al. 2020) through several approaches, such as resource/step selection functions (Davies et al. 2021), occupancy (Curveira‐Santos et al. 2021), activity pattern and overlap analyses (Rossa et al. 2021), and species distribution models (Jones et al. 2016). However, these data might also be used to investigate the co‐occurrence of an apex predator and a mesocarnivore at a broad scale (i.e., grid square system). In Europe, large carnivores' distributions are depicted through a 10 × 10 km grid square system, which provides a standardized method to map their presence (Chapron et al. 2014) and to highlight changes over the years (Kaczensky et al. 2024). Moreover, this grid‐based approach provides a standard method to summarize environmental conditions (e.g., landscape heterogeneity) and to compare them across the species' range (Frangini et al. 2025).

After a dramatic global decline due to human activities, the gray wolf *Canis lupus* (hereafter, wolf) survived with small and fragmented populations at the end of the 20th century (Ripple et al. 2014; Chapron et al. 2014). However, thanks to several conservation efforts worldwide (Boitani 2003), many populations have increased (Chapron et al. 2014), with one of the greatest recoveries observed in the Italian wolf population *C. l. italicus*. Indeed, from 100 individuals remaining in the central‐southern Apennines in the 1970s (Zimen and Boitani 1975), the population increased and reached the Alps in the 1990s (Valière et al. 2003; Fabbri et al. 2007), with a population estimate of 3307 individuals (95% CI 2,945–3,608) in 2020/2021 (La Morgia et al. 2022). The colonization of the Alpine range followed mainly a west‐to‐east direction, with the first sporadic appearances in the Eastern Italian Alps (i.e., Friuli Venezia Giulia Region, hereafter FVG) reported in 2013 (Marucco et al. 2018), and the first confirmed reproduction only in 2018 (Franchini et al. 2019). In the same area, the first sightings and reproductions of the golden jackal *Canis aureus* (hereafter, jackal) were reported in the 1980s (Lapini 2009–2010). This medium‐sized mesocarnivore has been showing a notable expansion in Europe and reached the FVG from the opposite dispersal direction compared to wolves (Spassov and Acosta‐Pankov 2019). In FVG, jackal population showed the highest densities in Italy, and from here it started to expand toward new areas of the Italian Peninsula, reaching approximately 150 individuals in 2020 (Franchini et al. 2019). Spassov and Acosta‐Pankov (2019) reported that the jackal distribution range was mainly limited to the southeastern part of the continent (i.e., the Balkans) until the 1970s, when it started to expand due to several factors. Among these factors, considerable evidence suggests that the jackal's expansion was favored by the disappearance of its natural competitor, the wolf (Krofel et al. 2017; Ranc, Wilmers, et al. 2022). Nonetheless, this potential effect is still debated. Jackals share most of their intercontinental range with wolves, and the suppression effect by the latter is most effective within wolf core areas (Newsome et al. 2017), with jackals potentially being part of the wolf's diet (Mohammadi et al. 2019). Therefore, a top‐down effect exerted by the wolf seems consistent, at least on a broad scale (Krofel et al. 2017; Ranc, Wilmers, et al. 2022). On the other hand, there is evidence of tolerance between the two species, which some authors hypothesized may have led to hybridization events (Moura et al. 2014; Stefanović et al. 2024). Indeed, in India, jackals have been observed using wolves' kill remains or even sharing food (Jhala 1993; Sazatornil et al. 2024). Since wolves may provide carcasses for scavengers, the trade‐off between the risk of wolf encounters and reward for food intake is context‐dependent (Diserens et al. 2022) and even mediated by social status (Atwood and Gese 2008). Therefore, at a smaller scale, there may be a facilitation process modulating spatio‐temporal segregation between these two wild canids. Few studies have investigated the jackal‐wolf interaction on a local scale and within a human‐dominated landscape (Krofel et al. 2017). Therefore, FVG represents a focal area to study intraguild interactions among these protected wild canids (Lapini and Rondinini 2013; Marucco et al. 2013), since it experienced first the colonization of the jackal and secondly the recolonization of the wolf (Filacorda et al. 2023). This study aimed to investigate the influence of the wolf on the jackal presence over 11 years (2013–2023) of monitoring. We hypothesize that the wolf's return may have exerted a top‐down effect on the jackal. Therefore, we would expect to observe a change in jackal occurrence over time, also in response to environmental characteristics, such as terrain ruggedness and habitat fragmentation. Specifically, heterogeneous, human‐dominated areas may favor the sympatric presence of the jackal and wolf, due to natural and anthropogenic food availability, as well as human presence that may be used as a shield to counteract the suppression effect by the wolf (Ranc, Wilmers, et al. 2022). The alternative hypothesis is that the wolf's presence may have favored the jackal by providing prey carcasses where the jackal could scavenge. To test our hypotheses, we: (i) compiled a year‐level dataset on the distribution of both species in the study period, highlighting also areas of sympatric presence and (ii) investigated whether there are environmental covariates explaining their distribution status at a broad (regional) scale. Moreover, we (iii) tested whether the presence of the wolf and other environmental covariates influenced the presence of the jackal at a finer (local) scale.

## 2 Materials and Methods

### 2.1 Study Area

**FIGURE 1:**
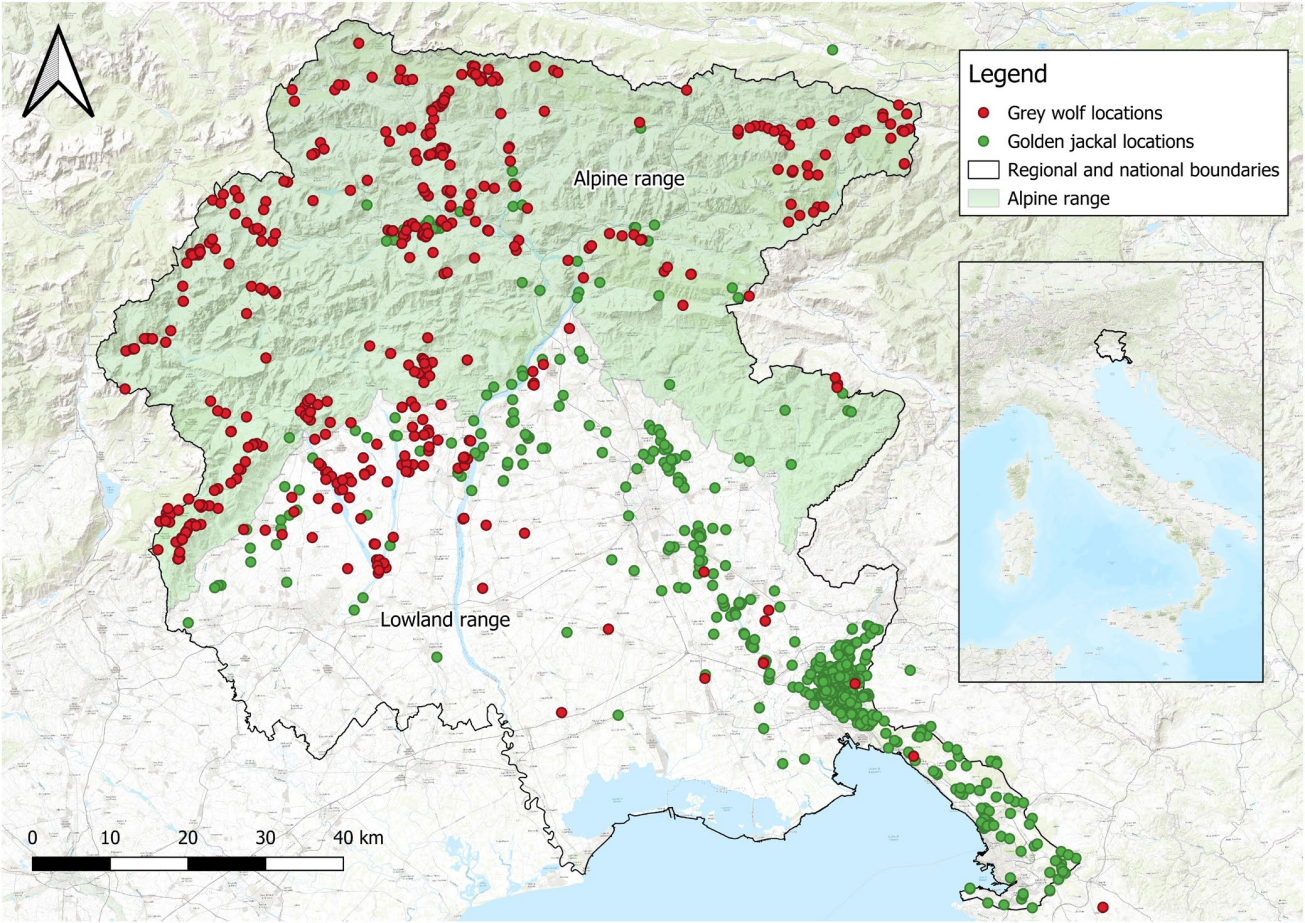
Friuli Venezia Giulia Region and species' locations (both C1 and C2) over the 2013–2023 period. The northern area is represented by the alpine range, characterized by a mountainous landscape, whereas the southern part is represented by the lowland range, characterized by a flat landscape with the karst (south‐eastern) as the only hilly district. Background represented by the ESRI World Topo (*Source:* ESRI).

The FVG is the most north‐eastern administrative region of Italy, covering 7920 km^2^. It borders Austria to the north and Slovenia to the east, and it faces the Adriatic Sea to the south and the Veneto region to the west (Figure 1). The region shows a great biological and ecological richness, determined by its location and geographical conformation (Poldini et al. 2006). FVG encompasses a wide range of habitats, varying from natural mountainous areas with few urban settlements and roads to heavily human‐modified landscapes with highways and high volumes of road traffic. These areas are classified into four ecoregions (Poldini et al. 2006): the Alpine and pre‐Alpine areas, located in the northern part of the region, are characterized by mountain landscapes dominated by forest and shrubland habitats. The southern part of FVG comprises the lowland ecoregion, which shows less vegetation cover, high levels of human activity, intensive agriculture, and near‐pristine rivers. The fourth ecoregion, the Karstland, lies in the southeastern part of FVG and consists of a hilly landscape dominated by broad‐leaved forests and anthropogenic *Pinus* sp. reforestations (Figure 1).

### 2.2 Data Collection

**FIGURE 2:**
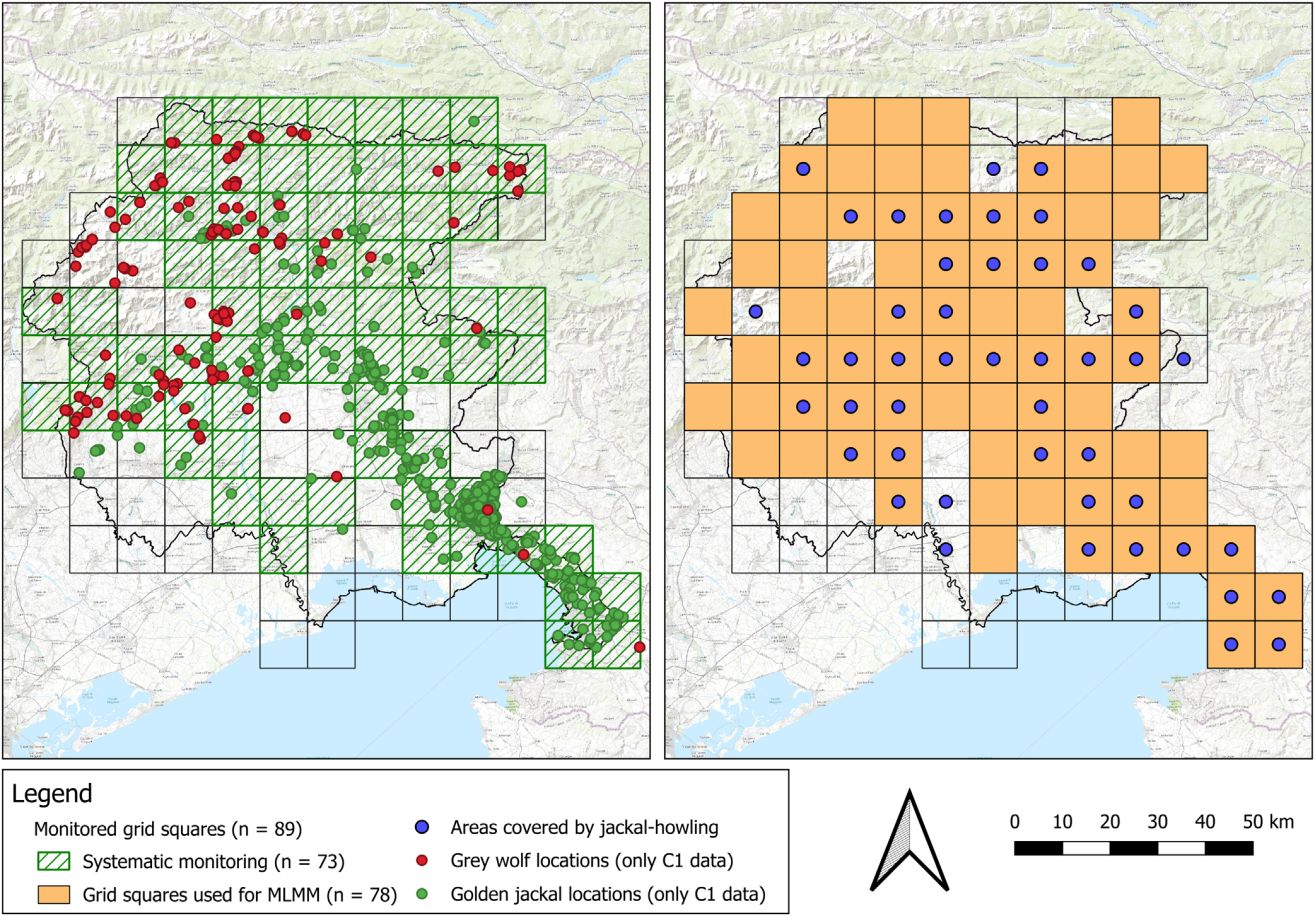
Monitored square grids. In the left panel, green squares were investigated through systematic monitoring techniques. Red and green dots represent C1 data for wolf and golden jackal, respectively. In the right panel, orange square grids represent sampling units analyzed through the multinomial logistic mixed model (MLMM). Blue dots represent square grids where jackal presence was investigated through jackal‐holwling and analyzed through the generalized linear mixed model (GLMM). Background represented by the ESRI World Topo (*Source:* ESRI).

We investigated the presence of jackal and wolf in 2013–2023, dividing FVG into 10 × 10 km UTM grid squares (Chapron et al. 2014). We collected species' occurrences through systematic surveys integrated with opportunistic data (Figures 1 and 2). Systematic surveys were carried out by the University of Udine and the Regional Administration of FVG with different techniques: camera‐trapping on both species, acoustic stimulations only for the jackal, and transects for the wolf only. Opportunistic data included carcass collection (e.g., road‐killed animals), signs of presence (scats, predations, tracks), direct observations, and IUCN data only for the jackal (Ranc, Acosta‐Pankov, et al. 2022). We covered 89 grid squares with both systematic and opportunistic data: while 73 grid squares were covered with systematic surveys, all 89 grid squares were covered with opportunistic data (Figure 2, left panel).

We briefly describe the systematic survey techniques and the associated sampling effort. For jackal acoustic stimulation (hereafter, jackal‐howling), we followed the protocol suggested by Comazzi et al. (2016): a prerecorded howl from a specific location (calling station) is emitted to induce a response that is used by the operators to estimate the distance, azimuth, and number of individuals (Giannatos et al. 2005; Frangini et al. 2022). Since the distance is the most difficult parameter to estimate, the operators were trained to measure it using empirical trials through a 100 m step approach (e.g., 100 m, 200 m, etc.) before field surveys. Calling station locations were selected to minimize the potential background noise from anthropogenic factors (e.g., main roads, towns, airports), and were located at least two kilometers away from each other. Specifically, we investigated 57 grid squares with jackal howling, with a total number of 201 independent calling stations repeated on average 2 times (min = 1, max = 21) in 2013–2023 (yearly average of 5 calling stations per grid square). Camera‐trapping data were collected during different research activities. In summary, we deployed camera traps to detect medium and large carnivores within historical areas of species presence, as well as within new areas that may be colonized. Most cameras were deployed along forestry roads, hiking paths, and wildlife trails without baiting for 1–2 months before being moved to another location (minimum distance of 100 m). Overall, we covered 43 grid squares with 448 camera‐trap locations (on average 10 camera‐traps per grid square), for a total amount of 52,126 camera‐trap nights (yearly average 467 camera‐trap nights per grid square) in the period 2014–2023. Transects were carried out mainly during Alpine‐scale surveys in 2014–2018 and 2020–2023, from October to April along forestry roads and hiking trails looking for signs of presence (scats, tracks, and predations) (Marucco et al. 2018; Marucco et al. 2022; Marucco et al. 2023). Overall, we conducted 457 transects across 3,797 km, covering 57 square grids, with an average of 32 transects per year that were repeated 1 to 7 times at each monitoring year (yearly average distance covered of 13.31 km per grid square).

As specified above, opportunistic data came from multiple sources: carcass occurrences (mainly road‐killed individuals) of jackal and wolf were retrieved from the progressive web application InfoFaunaFVG (Tomè et al. 2023). For the wolf, every opportunistic scat, track, predation, or direct observation was recorded and verified by trained operators. Lastly, since opportunistic data on the jackal were not recorded extensively as done for the wolf, we used IUCN presence data from Ranc, Acosta‐Pankov, et al. (2022) to integrate our presence maps (see next paragraph).

All data were classified according to the SCALP (Status and Conservation of the Alpine Lynx Population) criteria (Molinari‐Jobin et al. 2011) to ascertain the correct species identification and assess the data reliability (Molinari‐Jobin et al. 2012). Therefore, data were classified into three categories: C1, representing “hard evidence”; C2, “confirmed observations”, validated by experts; C3, “unconfirmed observations” not validated (Molinari‐Jobin et al. 2012; Marucco et al. 2020). As suggested by Marucco et al. (2022), we removed all the C3 occurrences to avoid biases in the analyses.

### 2.3 Statistical Analyses

To test for temporal and environmental covariates likely explaining the distribution status of the species at a broad scale, i.e., “Absence,” “Wolf presence,” “Jackal presence,” and “Sympatry”, we fitted a multinomial logistic mixed‐effect model (MLMM), using each grid square as a sampling unit and the grid square ID as the random effect. To maintain a conservative approach, grid square annual presence categories were based only on C1 data and relative to the sampling year: “Absence” was defined when no data were available, “Sympatry” when both species were detected, and “Wolf presence” and “Jackal presence” when only one of the two species was detected. Temporal covariates are represented by the sampling year (Year); meanwhile, landscape covariates were: ecoregion where the grid square was located (Alpine/Lowland; Area) (Figure 1), average terrain ruggedness index (TRI), landscape division index (LDI), and three metrics taking into account the anthropic patches, namely the percentage of landscape (PLand), Euclidean nearest neighbor (ENN), and the number of patches (NP). LDI, PLand, ENN, and NP were calculated for each grid square through the ‘landscapemetrics’ R package (Hesselbarth et al. 2019) using a high‐resolution land cover map from which we extracted anthropic patches (Marsoner et al. 2023). We tested for multicollinearity through the Variance Inflation Factor (VIF), removing covariates that showed VIF values greater than 5 (Zuur et al. 2010). Residual plots of some predictors showed clear non‐linearity, and we therefore fitted a non‐linear MLMM using natural splines for TRI (df = 3), LDI (df = 2), and Year (df = 2), choosing those parameters to meet model assumptions (Schuster et al. 2022). To highlight how the areas occupied by both species changed throughout the study period and to discuss our results, we plotted the number of occupied grid squares and their relative environmental covariates year by year.

Secondly, we tested for the wolf influence, along with other covariates, on the presence of jackal at a finer scale. Since jackal howling was the only systematic monitoring method with consistent sampling effort throughout each year of the study period, we compared the positive calling stations (1) vs. the negative ones (0) through a generalized linear mixed effect model (GLMM), setting the calling station as the random effect. To account for the unequal sampling effort, which was greater in the lowland, we used Binomial GLMM for proportional data (Zuur et al. 2009). To avoid autocorrelation among calling stations, we excluded those investigated on the same night closer than 4 km, and we created a 2 km buffer around each calling station where we calculated environmental metrics (Šalek et al. 2013). We tested for a subset of the metrics cited above (i.e., average TRI, PLand, and LDI), adding a binomial covariate (Wolf) indicating whether the wolf was detected within the buffer in the same year of the stimulation. We tested for multicollinearity through the Variance Inflation Factor (VIF), removing covariates showing VIF values greater than 5 (Zuur et al. 2010). Moreover, we tested for model assumptions through the ‘DHARMa’ R package (Hartig and Hartig 2017). We tested for several model combinations and ranked them based on the AIC (Akaike 1974) and ΔAIC (Burnham and Anderson 2002). In the presence of models showing ΔAIC < 2, we performed model averaging by calculating Akaike's weight (*ωi*) (Burnham and Anderson 2002). Model fitting and selection were performed with the “MuMIn” R package (Barton 2017). After testing model combinations, we fitted the best model using natural splines for TRI (df = 5) and NP (df = 3) due to the non‐linearity of these predictors (Schuster et al. 2022).

## 3 Results

**FIGURE 3:**
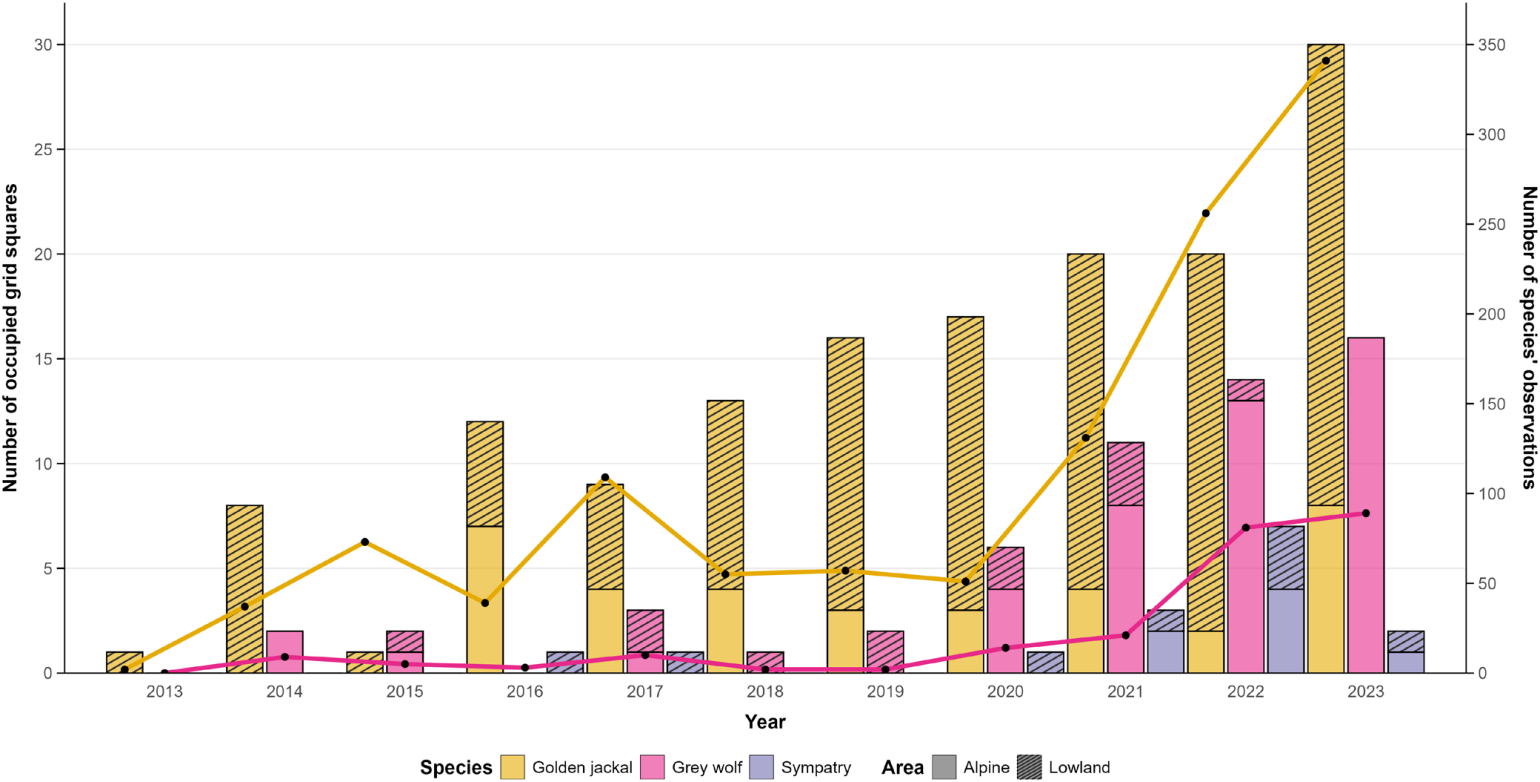
Number of grid squares occupied and number of C1 data across years. Bar plots represent the number of grid squares occupied by wolf, jackal, and both species (i.e., sympatry) divided by area (i.e., Alpine and lowland range depicted in Figures 1 and 2). Lines represent the number of C1 data collected for each species over the years.

**FIGURE 4:**
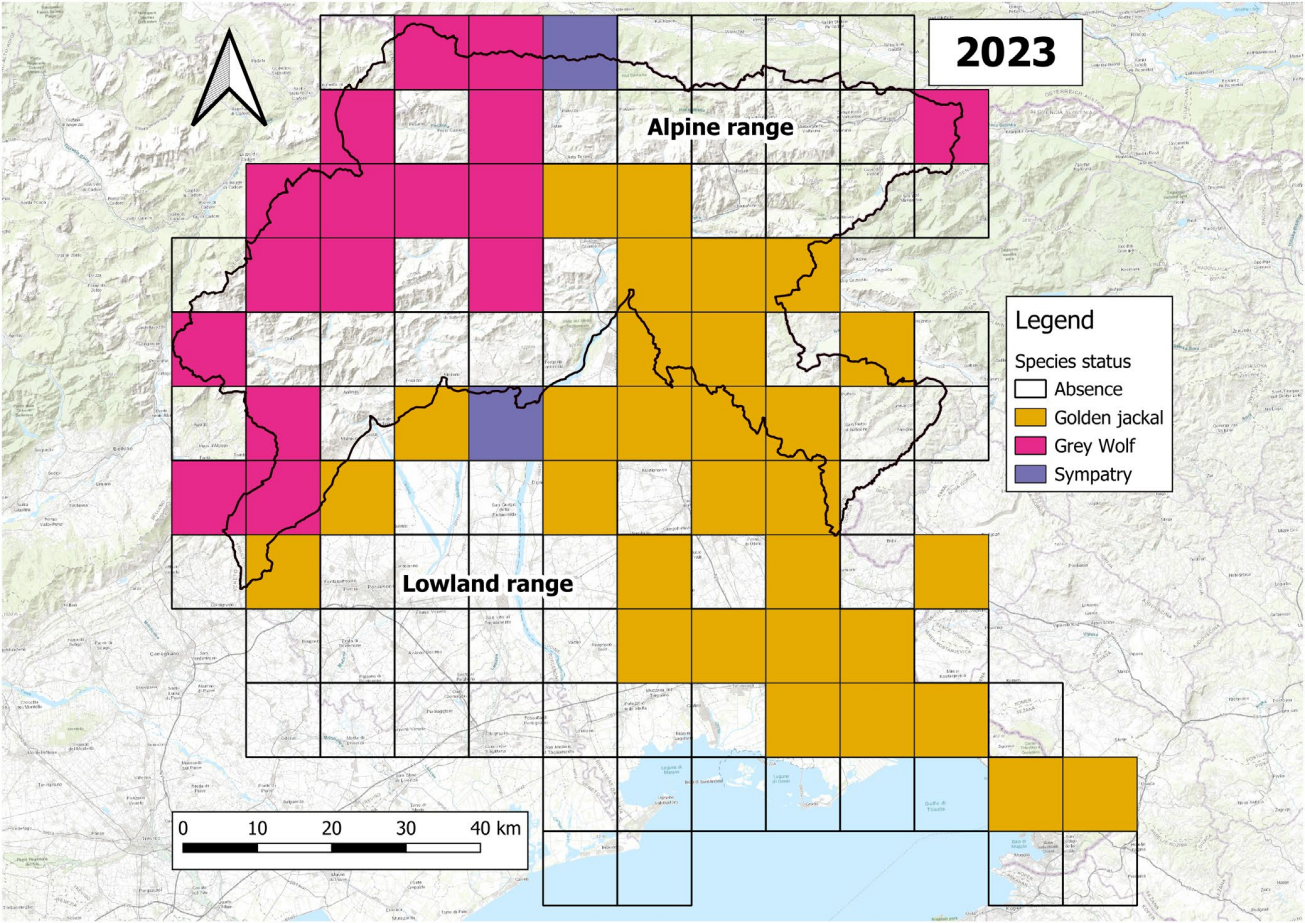
Species' distribution in 2023. The distribution map was generated using C1 data, and presence was assessed if at least one C1 occurrence lay within the square grid. Background represented by the ESRI World Topo (*Source:* ESRI).

**FIGURE 5:**
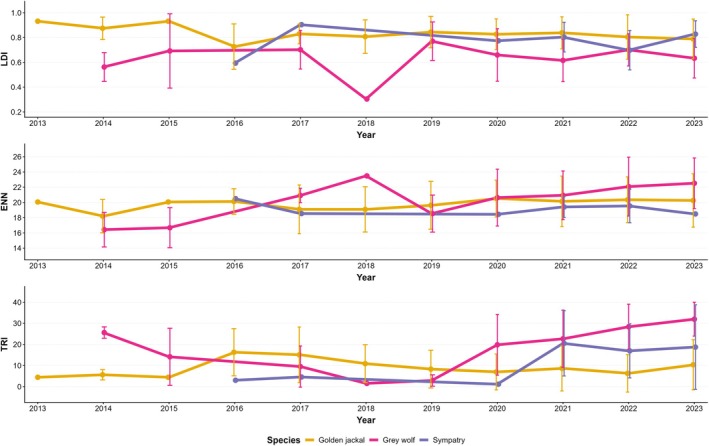
Yearly variations in the environmental covariates across the grid squares occupied by wolf, jackal, and both species (i.e., sympatry). Environmental covariates are: Landscape division index (LDI), Euclidean nearest neighbor (ENN), and Terrain ruggedness index (TRI). Values represent an average across all the grid squares for each category in the relative year; meanwhile, bars represent the Stardand Deviation.

**FIGURE 6:**
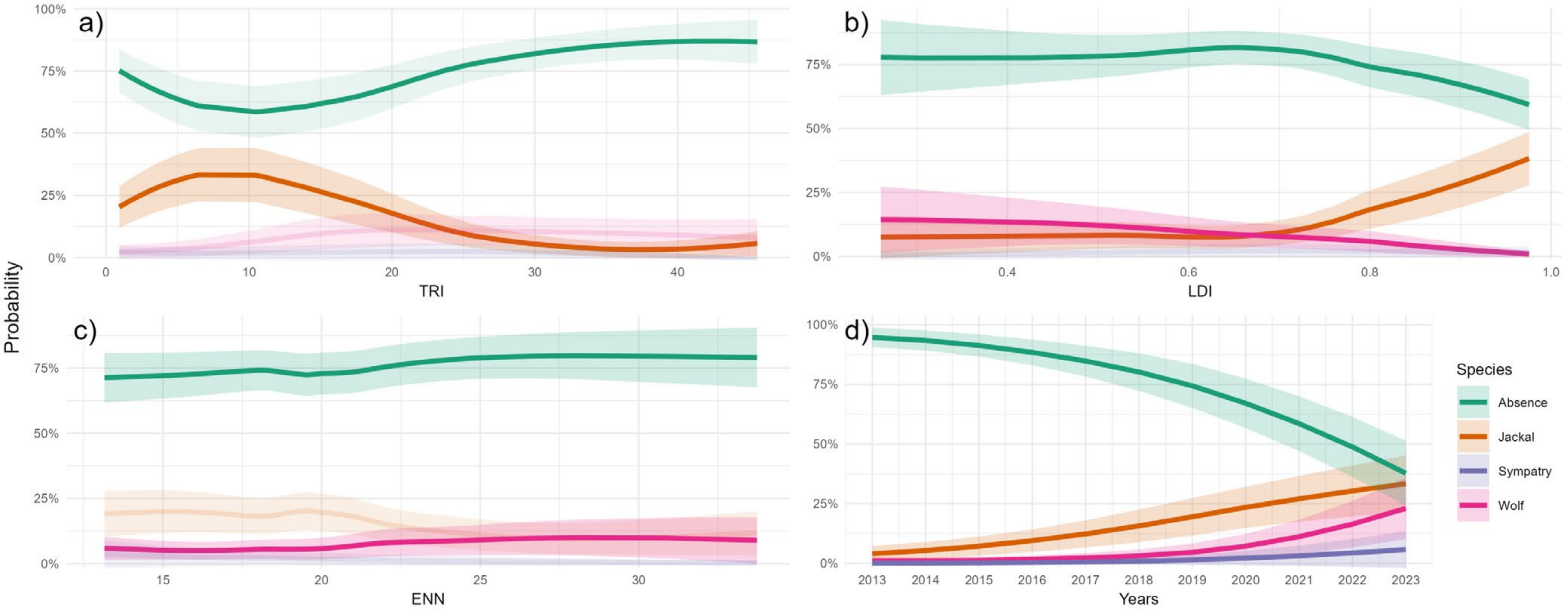
Predicted probabilities from the non‐linear MLMM of the covariates resulted in influencing the presence of species, i.e., (a) TRI (Terrain ruggedness index), (b) LDI (Landscape division index), (c) ENN (Euclidean nearest neighbor), and (d) the Year of sampling. For visualization purposes, shaded curves represent non‐significant covariates for the relative category of the response variable in the linear model. The only exception is panel (c), since ENN turned out to be significant only in the model with splines, and not in the linear one.

Overall, we obtained 1151 (*n*_C1_ = 1151) observations for the jackal and 661 (*n*_C1_ = 236, *n*_C2_ = 425) for the wolf (Figure 3). The number of occupied grid squares increased for both species, more linearly over the years for the jackal (*n*_2013_ = 1, *n*_2023_ = 30), while the wolf showed a net increase from 2020 (*n*_2013_ = 0, *n*_2023_ = 16), as did the sympatric grid squares (*n*_2013_ = 0, *n*_2023_ = 2) (Figures 3, 4, 5 and 6d). Except for 2016, the grid squares occupied by the jackal were always prevalent in the lowland, with a net increase across the study period, in contrast to the wolf, which exhibited the opposite pattern (Figure 3).

When testing for the species presence at the broad scale (i.e., square grid; *N*_unique grids_=78, *N*_unique grids ×11years_ = 858), we removed PLand and NP from the analysis due to multicollinearity issues, as well as “Area” due to model convergence issues. The multinomial mixed model (MLMM) with linear predictors indicated that Year had a statistically significant positive association with all categories relative to the reference level (‘Absence’). LDI was positively associated with jackal presence and negatively with wolf presence, while TRI showed a significant negative effect only on jackal presence (Table 1 and Figure 6). However, the non‐linear MLMM yielded a better model fit (ΔAIC = 10.27), with TRI and ENN gaining statistical significance for the wolf, and LDI losing statistical significance for both species (Table 1). Despite this, the predicted response curves revealed a clear non‐linear pattern for LDI, suggesting an effect that may not be well captured by a linear term (Figure 6). The probability of jackal presence showed a non‐linear pattern, with higher probabilities at low TRI and high LDI values, whereas wolf presence showed slightly higher probabilities at medium to high TRI values and lower probabilities at high LDI values. All categories showed increasing probabilities of presence across the study period, with the highest one for jackal, followed by wolf, and a slight increase in sympatry (Figure 6).

**TABLE 1:** Multinomial logistic mixed models (MLMMs) estimates, standard errors, and *p* values. The first box represents the linear model; meanwhile, the second one represents the non‐linear model, i.e., the one with natural splines. Variables statistically significant (i.e., *p* < 0.05) are denoted by “*”.

	Category vs. reference (“absence”)	Covariates	Estimate	SE	*Z*	*p*
Linear model	Jackal	Intercept	−1.91213	0.12968	−14.746	< 0.001*
		LDI	0.36513	0.12796	2.854	0.004*
		ENN	−0.084	0.112	−0.758	0.449
		TRI	−0.671	0.136	−4.932	< 0.001*
		Year	0.987	0.116	8.514	< 0.001*
	Sympatry	Intercept	−4.559	0.479	−9.502	< 0.001*
		LDI	0.037	0.308	0.121	0.904
		ENN	−0.376	0.323	−1.162	0.245
		TRI	−0.281	0.329	−0.854	0.393
		Year	1.645	0.402	4.094	< 0.001*
	Wolf	Intercept	−3.190	0.243	−13.103	< 0.001*
		LDI	−0.386	0.150	−2.580	0.010*
		ENN	0.114	0.149	0.766	0.444
		TRI	0.132	0.167	0.791	0.429
		Year	1.500	0.208	7.197	< 0.001*
Non‐linear model	Jackal	Intercept	−3.606	0.775	−4.655	< 0.001*
		LDI ns1	0.529	1.314	0.402	0.688
		LDI ns2	0.702	0.431	1.628	0.103
		ENN	0.030	0.122	0.245	0.806
		TRI ns1	−2.116	0.782	−2.706	0.007*
		TRI ns2	0.097	0.768	0.126	0.900
		TRI ns3	−2.991	0.726	−4.122	< 0.001*
		Year ns1	3.807	0.794	4.794	< 0.001*
		Year ns2	2.527	0.312	8.089	< 0.001*
	Sympatry	Intercept	−7.949	2.821	−2.818	0.005*
		LDI ns1	1.778	3.608	0.493	0.622
		LDI ns2	−0.084	1.196	−0.070	0.944
		ENN	−0.202	0.348	−0.580	0.562
		TRI ns1	0.326	1.882	0.173	0.863
		TRI ns2	−0.980	2.196	−0.446	0.655
		TRI ns3	−2.451	1.936	−1.266	0.205
		Year ns1	6.268	4.042	1.551	0.121
		Year ns2	4.119	1.093	3.767	< 0.001*
	Wolf	Intercept	−4.456	1.000	−4.456	< 0.001*
		LDI ns1	−2.207	1.221	−1.807	0.071
		LDI ns2	−1.531	0.825	−1.857	0.063


		ENN	0.384	0.177	2.175	0.030*
		TRI ns1	0.743	0.909	0.816	0.414
		TRI ns2	2.267	1.242	1.825	0.068
		TRI ns3	−1.378	0.698	−1.976	0.048*
Year ns1		3.705	1.497	2.475	0.013*
Year ns2	3.694	0.471	7.837	< 0.001*

Abbreviations: ENN, Euclidean nearest neighbor among urban patches; LDI ns(number), natural spline (number) for landscape division index; LDI, Landscape division index; TRI ns(number), natural spline (number) for terrain ruggedness index; TRI, Terrain ruggedness index; Year ns(number), natural spline (number) for year; Year, year of sampling.

**FIGURE 7:**
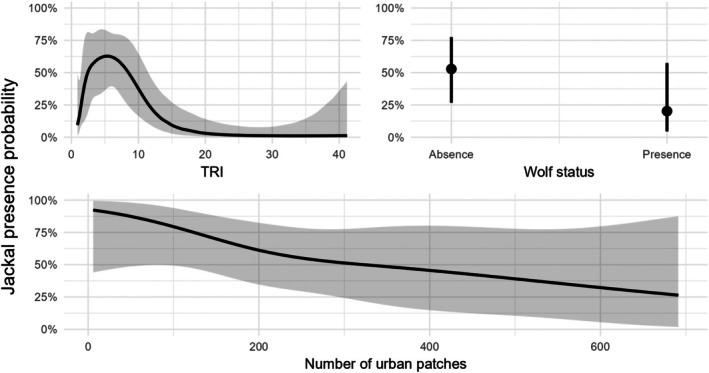
Predicted probabilities from the non‐linear GLMM of the covariates resulted in influencing the presence of the golden jackal, i.e., the wolf presence, the TRI (Terrain ruggedness index), and the NP (number of urban patches). Specifically, the jackal presence probability is lower with higher values of TRI and NP, and with the presence of the wolf.

We investigated 154 independent calling stations, with a different number of replications for each one, obtaining a presence/absence dataset of 491 observations. When testing the jackal status at the finer scale, we removed PLand from the analysis due to multicollinearity issues. We performed 32 model combinations: the best was represented by the one with single effects of wolf, NP, and TRI (Table 2). Here, all predictors were statistically significant with a negative association with jackal presence (Figure 7). However, the non‐linear GLMM yielded a better model fit (ΔAIC = 2.06), with NP losing statistical significance. Despite this, the predicted response curves revealed a clear non‐linear pattern for NP, suggesting an effect that may not be well captured by a simple linear term (Figure 7). The jackal presence was higher with low values of TRI, in the absence of the wolf, and decreased with higher abundance of urban patches.

**TABLE 2:** Generalized linear mixed effect models (GLMMs) ranking with the best model in *italics*. Variables statistically significant (i.e., *p* < 0.05) are denoted by “*”.

Model	df	logLik	AICc	ΔAIC	Weight
NP* + TRI* + Wolf*	*5*	*−232.23*	*474.68*	*0.00*	*0.407*
LDI + NP* + TRI* + Wolf*	6	−231.87	476.06	1.38	0.204
ENN + NP* + TRI* + Wolf*	6	−231.89	476.10	1.42	0.200

Abbreviations: ΔAIC, the difference with the AIC value of the best model; AIC, Akaike's information criterion; df, degrees of freedom; ENN, Euclidean nearest neighbor among urban patches; LDI, landscape division index; logLik, log‐likelihood; NP, number of urban patches; TRI, terrain ruggedness index; Wolf, wolf presence relative to wolf absence.

## 4 Discussion

Using 11 years of data collection, we evaluated the relative distribution of jackal and wolf in FVG, their dynamics, and possible interactions. We were able to observe changes in the distribution of both species during the study period and their associations with environmental factors at a broad scale, while we found evidence of a negative interaction between the jackal and the wolf at a finer scale. Studying their presence through a grid system allowed us to observe distributional patterns for both species, revealing different colonization dynamics: the jackal showed a steady increase in its range since the beginning of our study, whereas the wolf started only in the last years. Interestingly, sympatric areas did not increase at the same rate as the wolf, showing only a slight change toward the end of the study period. Both models provided complementary results that helped us to describe colonization dynamics of FVG in 2013–2023. Jackals were more likely to occur in areas with moderate to low TRI values and with high fragmentation, while the wolf showed an opposite trend, at least for the LDI. Such observations, along with the negative jackal‐wolf relationship observed at the finer scale, potentially suggest a top‐down influence of the wolf on the jackal presence, supporting our initial hypothesis.

Our data collection on jackal began (systematically) a few years before the first confirmed wolf reproductions, allowing us—at least through one monitoring technique—to follow the colonization process of the jackal both before and after the recolonization of the wolf. At the beginning of our study period, first wolf observations were limited to a single pair roaming in the Alpine range, which disappeared without any reproduction events (Marucco et al. 2018). Although reproduction was first confirmed in 2018, wolf presence began to be more widespread in FVG only in 2020 (Figure 3), when at least four potential reproductive nuclei were detected, mainly in the Alpine range (Menzano et al. 2023). Therefore, it is likely that any wolf influence on jackal may have started in those years. Despite the observed change in wolf occurrence, the jackal colonization showed no deflections, constantly increasing throughout the study period (Figure 3). It is noteworthy that sympatric areas only slightly increased, suggesting that the two canids mainly occupied different areas: the Alpine range for the wolf and the lowland/karstland for the jackal. During the recolonization process across the Italian Peninsula, the wolf first occupied forested areas with low human disturbance (Zanni et al. 2023), moving toward more fragmented and human‐dominated areas (Frangini et al. 2025). In contrast, the well‐documented ecological plasticity of the jackal (Šalek et al. 2013; Fenton et al. 2021) allows it to thrive even in agricultural areas near human settlements. In this scenario, the jackal likely colonized FVG through a north‐western direction (Spassov and Acosta‐Pankov 2019), firstly occupying large natural areas, namely the karstland and the Alpine range (Ranc, Acosta‐Pankov, et al. 2022), taking advantage of the absence of a larger competitor.

In the last years, the growing wolf population in the Alps (Marucco et al. 2023) has led to the recolonization of the FVG region, starting from the west and occupying continuous forested areas with low human disturbance, represented by the Alpine range (Menzano et al. 2023). The influence of the wolf on temporal and spatial patterns of other mesocarnivores has been widely described, especially on coyotes during the wolf recolonization in North America (Arjo and Pletscher 1999; Levi and Wilmers 2012; Klauder et al. 2021). At a broad scale, wolf presence is considered one of the main limiting factors for jackal distribution and expansion (Krofel et al. 2017), with possible lethal encounters for the jackal (Mohammadi et al. 2017). Therefore, in our study area, the wolf may have exerted a shift in the colonization of jackals toward flatter, fragmented areas close to human settlements (Fenton et al. 2021), where it can exploit anthropogenic food resources due to its high dietary flexibility (Ćirović et al. 2016; Lanszki et al. 2022). Such considerations are supported by the increase in the road‐killed jackals in FVG in the last period (Frangini et al. 2022; Tomè et al. 2023) as a consequence of the colonization shift, along with better data collection (Tomè et al. 2023) and a population increase (Frangini et al. 2022).

Our study represents a first step to investigate the influence of a top predator, the wolf, on a subordinate mesocarnivore, the jackal, in FVG and Western Europe. However, we urge caution in interpreting our results, as this study has important limitations, mainly due to unequal sampling effort across years, areas, and species, which may have biased our results. Moreover, assessing a species' absence using only one monitoring technique (e.g., jackal howling) might lead to false negative detections, particularly for carnivores that display nocturnal activity patterns and low population densities (Balme et al. 2009). However, we tried to reduce this bias through multiple replications (e.g., more jackal howling stimulations in the same calling station during different nights), as well as working on a broad scale (i.e., grid squares) to capture patterns of presence and their changes over time. Moreover, all confirmed wolf observations, whether systematically or opportunistically collected, are stored in a regional database, providing important insights into areas where the species is present. Although our work is correlational, model results suggest a top‐down effect exerted by the wolf, highlighting the need to further investigate this dynamic, which still relies on a few scientific evidences (Krofel et al. 2017; Newsome et al. 2017; Ranc, Wilmers, et al. 2022). Higher wolf densities seem to be a considerable factor in reducing mesocarnivore abundances (Berger and Gese 2007; Newsome and Ripple 2015; Newsome et al. 2017), and may affect the dispersal rate. Nonetheless, this influence seems not to strongly limit the extent of areas occupied by the jackal in our study area: after the increase of wolf packs in FVG, jackals continued to expand their range, especially in the lowland, reflecting their adaptability as a generalist mesocarnivore (Fenton et al. 2021). Moreover, within and nearby our study area, wolves' diet relies mainly on large herbivores (Buzan et al. 2024), while jackals prey on small rodents or scavenge on carcasses (Torretta et al. 2021; Hatlauf and Lanszki 2024). Therefore, since we observed just the initial process of recolonization of the wolf, it can be hypothesized that jackals will gradually optimize their behavior to mitigate the risk of wolf encounter (Creel et al. 2001) while taking advantage of wolves' kill remains (Atwood and Gese 2008). On the other hand, we cannot exclude that such behaviors are already occurring where these two wild canids live in sympatry. The absence of significant environmental covariates for sympatry may suggest that facilitation could occur in both Alpine and Lowland areas, but may be modulated by other parameters, such as species densities, rather than simple presence or absence.

## 5 Conclusions

Two distinct processes, namely the colonization of new areas by the jackal and recolonization by the wolf, have led these two wild canids to live in sympatry in Europe. One of the first areas in western Europe where this carnivore overlap has occurred is our study area, which can be considered a ‘natural lab’ to understand ecological relationships between these two species. Typically, such analyses can be performed through intensive systematic sampling schemes, which are not always feasible in terms of time and cost. Therefore, we integrated data from multiple sources to gain some insights about the ecological dynamics of these canids. Our data depicted an increase in distribution for both species and, to a lesser degree, for sympatric areas, and a negative relationship at a finer scale between these canids, all suggesting a top‐down suppression. The jackal colonization occurred mainly through less rough (TRI) and more fragmented landscapes (LDI), whereas the wolf showed an opposite trend. On the other hand, sympatric areas could not be clearly characterized in terms of environmental covariates. Fine‐scale environmental analyses reflected the results found at broad scale and provided further evidence of the negative influence of the wolf on the jackal. Nonetheless, the wolf presence does not seem to be a strong negative driver for jackal expansion in more fragmented areas but may reduce its capacity to expand and establish new packs in less human‐dominated landscapes, such as the Alpine range, where it's relegated mainly to the valley bottoms.

However, our work has some limitations, i.e., spatial and temporal biases in sampling occurrences which we tried to mitigate with different methods, as well as the opportunistic nature of some data, which may have biased some of our results. Since across FVG, many areas had not been constantly and systematically surveyed, and/or no opportunistic data had been collected, more systematic samplings should be performed to strengthen our results. Notwithstanding, our work represents a first step in studying the interactions between these competitive species in Italy. We emphasize the importance of future analyses, which could strengthen our results and should be based on more robust sampling schemes, integrate both demographic and behavioral parameters, and investigate dispersal rates to shed light on such complex interactions.

## Author Contributions

Lorenzo Frangini: conceptualization (lead), data curation (lead), formal analysis (lead), investigation (equal), methodology (lead), validation (lead), visualization (lead), writing – original draft (lead), writing – review and editing (lead). Lorenzo Bernicchi: conceptualization (lead), data curation (equal), formal analysis (equal), investigation (lead), methodology (equal), validation (equal), visualization (equal), writing – original draft (equal), writing – review and editing (equal). Marco Franchetto: data curation (equal), investigation (equal), writing – review and editing (equal). Ursula Sterrer: data curation (equal), investigation (equal), writing – review and editing (equal). Hanna Steigleder: data curation (equal), investigation (equal), writing – review and editing (equal). Matteo Canazza: data curation (equal), investigation (equal), writing – review and editing (equal). Marcello Franchini: data curation (equal), investigation (equal), writing – review and editing (equal). Virginia Barca: data curation (equal), investigation (equal), writing – review and editing (equal). Giovanna Miani: data curation (equal), investigation (equal), writing – review and editing (equal). Andrea Madinelli: data curation (equal), investigation (equal), writing – review and editing (equal). Stefano Filacorda: conceptualization (lead), data curation (equal), funding acquisition (lead), investigation (lead), project administration (lead), resources (lead), supervision (lead), writing – original draft (lead), writing – review and editing (equal).

## Conflicts of Interest

The authors declare no conflicts of interest.

## Data Availability

The data supporting the findings of this study are publicly available in the Dryad Digital Repository, at the following link: https://doi.org/10.5061/dryad.12jm63z8h.
